# Crosstalk Among Gut Microbiota, Fecal Metabolites, and Amygdala Neuropathology Genes After Ginger Polyphenol Administration in Female Rats with Neuropathic Pain: Evidence for Microbiota–Gut–Brain Connection

**DOI:** 10.3390/nu17091444

**Published:** 2025-04-25

**Authors:** Chwan-Li Shen, Julianna Maria Santos, Moamen M. Elmassry, Fang Chen, Guangchen Ji, Peyton Presto, Takaki Kiritoshi, Xiaobo Liu, Volker Neugebauer

**Affiliations:** 1 Department of Pathology, Texas Tech University Health Sciences Center, Lubbock, TX 79430, USA; julianna.santos@ttuhsc.edu (J.M.S.); xiaobo.liu@ttuhsc.edu (X.L.); 2 Center of Excellence for Integrative Health, Lubbock, TX 79430, USA; volker.neugebauer@ttuhsc.edu; 3 Center of Excellence for Translational Neuroscience and Therapeutics, Lubbock, TX 79430, USA; guangchen.ji@ttuhsc.edu (G.J.); peyton.presto@ttuhsc.edu (P.P.); 4 Department of Microanatomy and Cellular Biology, Woody L. Hunt School of Dental Medicine, Texas Tech University Health Sciences Center, El Paso, TX 79905, USA; 5 Department of Molecular Biology, Princeton University, Princeton, NJ 08540, USA; elmassry@princeton.edu; 6 Center for Biotechnology and Genomics, Texas Tech University, Lubbock, TX 79409, USA; che93458@ttu.edu; 7 Department of Pharmacology & Neuroscience, Texas Tech University Health Sciences Center, Lubbock, TX 79430, USA; takaki.kiritoshi@ttuhsc.edu; 8 Garrison Institute on Aging, Texas Tech University Health Sciences Center, Lubbock, TX 79430, USA

**Keywords:** ginger, neuropathic pain, gut microbiome, neuropathology, brain, rats

## Abstract

**Objectives.** The relationships among neuropathic pain, gut microbiota, microbiome-derived metabolites, and neuropathology have received increasing attention. This study examined the effects of two dosages of gingerol-enriched ginger (GEG) on mechanical hypersensitivity, anxiety-like behavior, gut microbiome composition and its metabolites, and neuropathology markers in female rats in the spinal nerve ligation (SNL) model of neuropathic pain. **Methods.** Forty female rats were assigned to 4 groups: sham-vehicle, SNL-vehicle, SNL+GEG at 200 mg/kg BW, and SNL+GEG at 600 mg/kg BW via oral gavage. All animals were given an AIN-93G diet for 5 weeks. Mechanical hypersensitivity was assessed by the von Frey test. Anxiety-like behavior was assessed by the open field test. Fecal microbiota composition and metabolites were determined using 16S rRNA gene sequencing and GC-MS, respectively. Neuropathology gene expression profiling of the amygdala was assessed by an nCounter^®^ Neuropathology pathway panel. **Results.** Both GEG-treated groups showed decreased mechanical hypersensitivity and anxiety-like behavior in the SNL model. Gut microbiome diversity in both GEG groups was decreased compared with untreated SNL rats. In the SNL model, phyla such as *Bacteroidota*, *Proteobacteria* and *Verrucomicrobiota* were decreased. Compared with the untreated SNL group, both GEG groups exhibited increased abundance of the phyla *Bacteroidota* (i.e., *Rikenella*, *Alistipes*, *Muribaculaceae*, *Odoribacter*), *Firmicutes* (i.e., *UBA1819*, *Ruminococcaceae*, *Oscillospiraceae*, *Roseburia*), and *Verrucomicrobiota* (i.e., *Victivallis*). GEG groups had higher levels of nine hydrophilic positive metabolites [val-glu, urocanic acid, oxazolidinone, L-threonine, L-norleucine, indole, imino-tryptophan, 2,3-octadiene-5,7-diyn-1-ol, and (2E)-3-(3-hydroxyphenyl) acrylaldehyde] and two hydrophilic negative metabolites [methylmalonic acid and metaphosphoric acid], as well as lower levels of five hydrophilic metabolites [xanthine, N-acetylmuramic acid, doxaprost, adenine, and 1-myristoyl-2-oleoyl-sn-glycero-3-phosphoethanolamine]. Among the 770 neuropathology genes, 1 gene (*PLA2G4A*) was upregulated and 2 genes (*CDK5R1* and *SHH*) were downregulated in SNL rats. GEG caused the upregulation of nine genes (*APC*, *CCNH*, *EFNA5*, *GRN*, *HEXB*, *ITPR1*, *PCSK2*, *TAF9*, and *WFS1*) and downregulation of three genes (*AVP*, *C4A*, and *TSPO*) in the amygdala. **Conclusions.** GEG supplementation mitigated pain-associated behaviors in female rats with neuropathic pain, in part by reversing the molecular neuropathology signature of the amygdala. This was associated with changes in the gut microbiome composition and fecal metabolites, which could play a role in mediating the effects of GEG on neuropathic pain.

## 1. Introduction

Neuropathic pain (NP) refers to pain affecting the somatosensory system due to a lesion or disease, such as damage to a peripheral nerve. NP affects 7–10% of people worldwide [1], and its mechanisms are not well understood.

The microbiota–gut–brain axis has received increased attention lately, with more information emerging about the role played by gut microbiota along with gut-derived metabolites in the development of NP. Gut microbiota have a critical role in regulating immune, neural, endocrine, and metabolic signaling pathways, which all interact with one another to affect NP progression. Gut microbiota activate nociceptors via their byproducts and derived metabolites in NP progression [2,3]. Modification of gut microbiota abundance and composition in the intestine can influence the peripheral nervous system (PNS) and central nervous system (CNS), resulting in modified brain functioning via the microbiota–gut–brain axis [3]. Microbiome-derived metabolites are also linked to NP development, since the gut microbiome interacts with the PNS or CNS through metabolites, immune mediators, and nervous structures [3].

Recent studies have shown beneficial effects of ginger bioactive compounds in modulating gut microbiota as well as NP [4,5]. Ginger (*Zingiber officinalis* Roscoe), a combination of gingerols, shogaols, and paradols, mitigates NP-induced pain and pain-induced anxiodepressive behavior in male rats, demonstrating the anti-inflammation and antioxidant capabilities of ginger [4,5,6]. In a spinal nerval ligation (SNL)-induced NP male model, we reported that supplementation of dietary gingerol-enriched ginger (GEG) significantly modulated the composition of gut microbiota in feces [4]. In the same male NP study, GEG supplementation resulted in increased levels of fecal metabolites with anti-inflammatory and antioxidant properties [4]. However, GEG’s effects on the composition of the gut microbiome and its derived metabolites in female NP rats are not yet known.

NP is connected to peripheral nerve injury, which triggers both neuronal and glial or non-neuronal components of the peripheral/central cellular circuitry. Additional neuro-glial interaction leads to central sensitization and pain hypersensitivity in NP progression [7]. Spinal nerve injury, a type of peripheral nerve injury, causes mechanical allodynia and an imbalance of neurotransmitters, leading to chronic pain, anxiety, and other disorders of the central nervous system [8]. Additionally, peripheral nerve injury may disrupt the barrier between the blood and the spinal cord, leading to an influx of peripheral immune cells. The monocyte chemoattractant protein-1 can mediate this effect [9,10]. Peripheral nerve damage produces a central inflammatory signal cascade of astrocytes and microglia that contributes to behavioral hypersensitivity in NP development [11].

The amygdala is key to processing the affective and emotional aspects of pain and pain regulation. Increased nociceptive processing in the spinal cord and PNS results in signals being transmitted to different brain regions through ascending pathways. The central nucleus of the amygdala (CeA) fulfills pain-related output impacts within the circuity of amygdala. Then, the laterocapsular division of the CeA (CeLC) is able to receive nociceptive messages via the parabrachial nucleus, contributing to pain-associated behavior modulation [12,13]. The amygdala lies at the interface between the emotional/affective and sensory/cognitive aspects of pain. The neuroplasticity in the CeA has been associated to acute and chronic pain-associated behavioral responses [12,14,15]. The role of non-neuronal elements in the amygdala in pain plasticity and pain regulation is only beginning to emerge, but impaired amygdala astrocytic signaling has been previously linked to hyperexcitability and NP symptoms [16]. We reported in the past that gingerol polyphenols improved neuroimmune signaling in the amygdala of male NP rats [17].

The molecular mechanisms underlying ginger’s effects in NP in female rats have not yet been explored. In this study, we examined how GEG supplementation affects mRNA expression in the amygdala of female NP rats to identify the genes involved in GEG’s neuroprotection both in pain-associated behavior and at the molecular level. To achieve this, we utilized an nCounter^®^ targeted neuropathology transcriptome analysis from NanoString, Seattle, WA, USA. We specifically selected the nCounter^®^ neuropathology panel (770 genes) to study GEG’s effects on the specific sets of genes involved in neurodegenerative diseases. The nCounter^®^ neuropathology panel offers a comprehensive evaluation of 23 neurodegenerative pathways/processes and profiles five types of core cells (neurons, astrocytes, microglia, oligodendrocytes, and endothelial cells) [18] that are involved in five themes (neurotransmission; neuroinflammation; metabolism; neuroplasticity, development, and aging; and compartmentalization, structure, and integrity).

There is much evidence to suggest that sex is an important factor in modulating pain. Data in the literature strongly suggest that men and women differ in their responses to pain: women report higher pain sensitivity and more common instances of painful diseases than men [19]. Factors influencing pain sensitivity in males and females include different modulation of the endogenous opioid system, neuroimmune signaling, and sex hormones [19]. For instance, in a rat model of partial-sciatic-nerve-ligation-induced NP, female rats were significantly more susceptible to developing hypersensitivity than male rats [20,21]. In a mouse model of chemotherapy-induced NP, cold allodynia, but not mechanical allodynia, was more robust in female mice treated with paclitaxel [22], mediated by differential circulating levels of sex hormones and inflammatory signals [23]. Thus, the investigation of treatment effects in female rats for NP management is well-justified.

This is not to negate the beneficial effects of ginger bioactive compounds in modulating gut microbiota and NP in male rats [4,5]. Ginger (*Zingiber officinalis* Roscoe), which contains a combination of gingerols, shogaols, and paradols, mitigates NP-induced pain and pain-induced anxiodepressive behavior in male rats, demonstrating the anti-inflammation and antioxidant capabilities of ginger [4,5,6]. In the spinal nerval ligation (SNL)-model of NP in male rats, we reported that the supplementation with dietary gingerol-enriched ginger (GEG) significantly mitigated pain-associated behavioral responses and modulated the composition of gut microbiota in feces [4]. In the same study, GEG supplementation resulted in increased levels of fecal metabolites in male rats because of its anti-inflammatory and antioxidant properties [4]. However, GEG’s effects on the composition of the gut microbiome and its derived metabolites in female NP rats are not yet known, which is the basis for the current study. To the best of our knowledge, no known study has evaluated GEG’s impacts on NP symptoms, gut microbiota, microbiome-derived metabolites, and neuropathology genes in female NP animals. Therefore, we measured NP symptoms, gut microbiome abundance, composition, and derived metabolites and neuropathy genes in females with NP, as well as the effects of GEG administration on the animals. Our hypothesis stated that supplementation with GEG would decrease NP-associated mechanical hypersensitivity and anxiodepressive behavior in a dose dependent pattern through (i) modulating the composition of the gut microbiome with a greater abundance of beneficial microbiota, (ii) alteration of fecal metabolites, and (iii) alteration of gene expression within five themes of neuropathology. Our goal is a better understanding of ginger polyphenols’ impacts on metabolic and molecular pathways related to NP in order to devise a nutraceutical approach to manage NP.

In the present study, we used GEG rich in gingerols (~24% gingerols, especially 6-gingerol), which is a much higher concentration of gingerols than in standard ginger extract (~5% gingerols) [24]. Studying GEG allowed us to focus on the key bioactive compounds (gingerols) to better understand how gingerols contribute to neuroprotective effects in the neuropathic model studied here.

## 2. Materials and Methods

### 2.1. Animals, NP Induction, and Group Treatments

Forty female SD rats (5 wk old) were purchased from Envigo, Cumberland, VA, USA and individually housed under a 12 h light/dark cycle. The rats had access to water and AIN-93G diet ad libitum. After five days of acclimation, 10 rats received sham surgery and 30 rats received SNL surgery on the left L5 [17]. The day after sham or SNL surgery, all animals were assigned to: the Sham-V group, receiving corn oil; the SNL-V group, receiving corn oil; the SNL+200GEG group, receiving GEG at 200 mg/kg body weight; and the SNL+600GEG group, receiving GEG at 600 mg/kg body weight. Both corn oil and GEG were given via daily oral gavage for the 4-week study intervention. An AIN-93G diet was purchased from Research Diet Inc., New Brunswick, NJ, USA. The GEG was a gift from Sabinsa Corporation, East Windsor, NJ, USA. The GEG consisted of 18.7% 6-gingerol, 1.81% 8-gingerol, 2.86% 10-gingerol, 3.09% 6-shogoal, 0.39% 8-shogaol, and 0.41% 10-shogaol [4,5,6]. This study was approved by the Institutional Animal Care and Use Committee (protocol number: 20032). We recorded food intake, water consumption, and body weight of animals weekly.

Our previous studies indicated that to obtain significance at the α = 0.05 at statistical power 0.9, n = 6–10 rats per group [5,6,7] are necessary to detect differences in mechanosensory thresholds, anxiety-like behavior, microbiome, and metabolites. Thus, in this study, we used 10 rats per group for the outcome measures.

### 2.2. Mechanical (Hyper)Sensitivity Measurement

At 1 day before surgery and weekly after surgery, we measured mechanical withdrawal thresholds of spinal nocifensive reflexes on the left rear paw following our previous study protocol [5]. Six measurements per subject were used for average mechanical hypersensitivity. To ensure consistency of behavioral testing, the same person carried out all assessments but was blinded to the group assignment. The test was performed in the same environment at the same time each day.

### 2.3. Anxiety-like Behavior Measurement

Prior to surgery and sample collection, we measured anxiety-like behavior using open field test (OFT) based on our published work [25,26]. We calculated the number of entries and the duration in the center area during the first 5 min [25,26]. To ensure consistency of behavioral testing, the same person carried out all assessments but was blinded to the group assignment. The test was performed in the same environment at the same time each day.

### 2.4. Sample Collection

At the end of study, the animals were anesthetized under isoflurane and euthanized for sample collection. The amygdala (central nucleus of the right hemisphere) and feces from the cecum were harvested, snapped in liquid nitrogen, and stored at −80 °C freezer for later laboratory work.

### 2.5. Gut Microbiota Profiling via 16S rRNA Amplicon Sequencing

We extracted DNA from feces using a DNA extraction kit (QIAamp PowerFecal Pro DNA kit, Qiagen Inc., Germantown, MD, USA). Molecular Research LP (Shallowater, TX, USA) conducted amplicon 16S rRNA sequencing based on our published studies [3,5,9]. The raw sequencing data (BioProject access number PRJNA935472) was deposited in the National Center for Biotechnology Information BioProject database.

### 2.6. Hydrophilic Metabolites in Feces

Fifty milligram fecal samples were homogenized in PBS buffer with a bead beater. After centrifugation (15,000 rpm, 10 min), the supernatant (100 µL) was collected and extracted with dichloromethane + methanol + H_2_O (1:2:1 ratio) on ice. Then, the mixture was vortexed and centrifuged again (5000 rpm, 15 min) to collect the aqueous layer. The aqueous layer was dried by vacuum (Speed VAC) and resuspended in 100 µL methanol + H_2_O (1:1) and subjected to analysis using ultrahigh-performance liquid chromatography–mass spectrometry (UHPLC-MS/MS). We applied mobile phase A (0.1% formic acid in H_2_O) and mobile phase B (0.1% formic acid in methanol) with a flow rate of 0.45 mL/min. The UHPLC gradients were 0–5.5 min, 0.5% to 50% B; 5.5–6 min, 50% to 98% B; 6–12 min, maintaining 98% B; 12–13 min, 98% to 0.5% B; 13–15 min, 0.5% B. We used a UPLC column (Waters Acquity, HSS T3, 1.7 µm, 2.1 mm × 100 mm) to separate the metabolites. The column temperature was set at 50 °C with a 10 μL sample injection volume. We employed a Q-Exactive HF mass spectrometer method to analyze both nontargeted positive and negative metabolites. The resolution was set at 120,000, the AGC target was 3e6, and the maximum IT was 200 ms. We used Compound Discoverer Software (Compound Discoverer ™ 3.3, Thermo Fisher Scientific, Waltham, MA, USA) to identify and quantify metabolites.

### 2.7. Gene Expression Profiling Using a Neuropathology Panel

We extracted total RNA from the amygdala (Qiagen RNeasy Mini Kit, Qiagen Inc., Germantown, MD, USA), measured total RNA concentration (Nanodrop 2000, Thermo Fisher Scientific, Waltham, MA, USA), and kept total RNA in a −80 °C freezer until later use. We performed RNA gene profiling of the amygdala with the nCounter^®^ neuropathology pathway panel (NanoString Technologies, Seattle, WA, USA) at the Department of Inflammation and Immunity, Cleveland Clinic, Cleveland, OH. Profile expression of neuropathology panel included 770 genes, including 757 genes in neuropathology-related core pathways and 13 housekeeping genes.

We selected NanoString’s nCounter Neuropathology Panel for the following reasons: (i) this panel provides a perspective on neurodegenerative processes by analyzing a wide range of genes within the six fundamental themes (neurotransmission, neuroglial interaction, neuroplasticity, cell structural integrity, neuroinflammation, and metabolism); (ii) the targeted genes address aspects of neurological disorders and injuries; (iii) this panel is commonly used in biomarker identification, drug treatment response studies, and mechanisms of neurodegenerative diseases; and (iv) this nCounter technology can directly quantify RNA abundance and offer acute and reproducible measurement without a need for amplification of RNA.

### 2.8. qRT-PCR Confirmation of Neuropathology Genes

We conducted qRT-PCR to validate the gene expression obtained from the neuropathology gene panel: APC (adenomatosis polyposis coli), CCNH (cyclin H), EFNA5 (ephrin A5), GRN (granulin), ITPR1 (inositol 1,4,5-trisphosphate receptor 1), PCSK2 (proprotein convertase subtilisin/kexin type 2), TAF9 (TATA-box binding protein associated factor 9), and WFS1 (Wolfram syndrome 1 homolog). Corresponding primers (Table 1) were used based on our published method [5,9]. All gene expression levels were normalized to our control, β-actin. A formula of 2 − (ΔCT × 1000) [27] was used to calculate the level of gene expression.

### 2.9. Statistical Analysis

The von Frey Test (VFT), OFT, and gene expression data are presented as the mean ± standard error of the mean (SEM). We checked data normality (Gaussian distribution) before conducting ANOVA. VFT, OFT, and RT-PCR data were analyzed by one-way or two-way ANOVA and then Bonferroni multiple comparison tests. We used the GraphPad Prism software (v9, San Diego, CA, USA) for data analysis and set a *p*-value < 0.05 as a significant difference. For neuropathology gene profiling analysis, we analyzed raw datasets using ROSALIND^®^ platform and performed enrichment analysis following our published work [28]. We also performed 3 pairwise comparisons (SNL-V vs. Sham-V, SNL+200GEG vs. SNL, and SNL+600GEG vs. SNL). For microbiome analysis, we filtered, denoised, and merged the reads, then analyzed 16S rRNA gene sequencing data using QIIME 2. Average sequencing depth was about 524,000 reads, and approximately 51,000 reads were retained after high-quality filtering. We used DADA2 to determine the exact amplicon sequence variants (ASVs). Taxonomy assignment was performed using the Greengenes database, version 13.8. For redundancy analysis, we used the Calypso software 64-bit version (Calypso Sofr Inc., Naperville, IL, USA) which generated a redundancy analysis (RDA) plot and measured the statistical significance of the group effect on microbial community composition. To compare the relative abundance of taxa between two groups, we conducted a compositional analysis using LOCOM 6.0 (LOgistic COMpositional) with false discovery rate control. *p*-value < 0.05 indicates significant difference. For fecal metabolites analysis, we performed ANOVA followed by the Benjamini–Hochberg procedure and *t*-test for pairwise comparisons and the Benjamini–Hochberg procedure for false discovery rate correction. Metabolites with *p*-value ≤ 0.001 were considered statistically significant.

## 3. Results

### 3.1. GEG Administration Mitigated Mechanical Hypersensitivity

**Figure 1:**
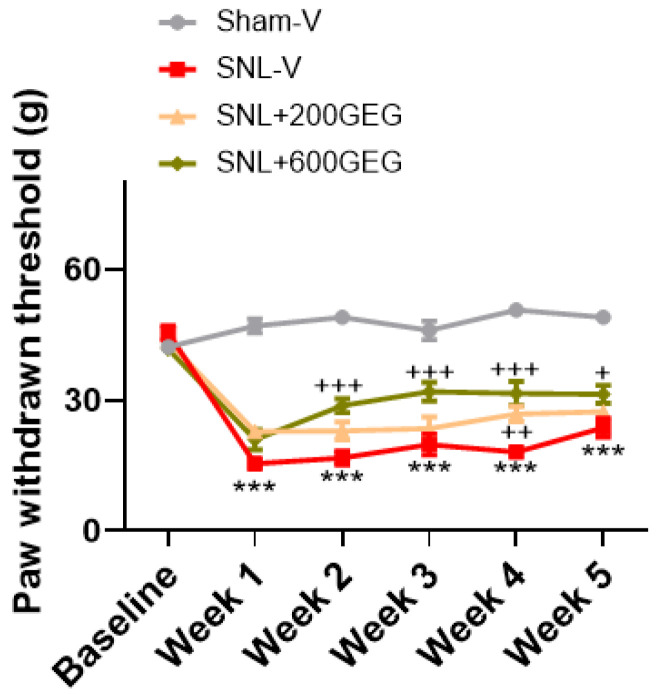
Effects of GEG on mechanosensitivity assessed by an electronic von Frey anesthesiometer. Data are expressed as mean ± SEM and were analyzed by two-way ANOVA with the Bonferroni multiple comparison test, n = 9 per group. *** *p* < 0.001 compared with Sham-V group. + *p* < 0.05, ++ *p* < 0.01, +++ *p* < 0.001 compared with SNL-V group.

We assessed GEG supplementation’s effects on NP-associated mechanosensory behavior using VFT (Figure 1). At baseline, no difference in mechanosensitivity was observed among all groups. Compared with the Sham-V rats, the SNL-V rats had greater mechanical sensitivity at 1 week after surgery and remained so for the duration of the study. At the study’s end, the SNL+600GEG rats, but not the SNL+200GEG rats, had significantly decreased hypersensitivity compared with the SNL-V rats (Figure 1).

### 3.2. GEG Administration Alleviated Anxiety-like Behaviors

**Figure 2:**
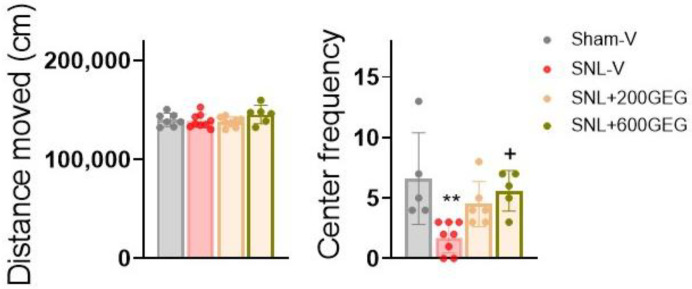
Effects of GEG on anxiety-like behavior assessed in the open-field test (OFT). Data are expressed as the mean ± SEM and were analyzed by one-way ANOVA with the Bonferroni multiple comparison test, n = 5–8 per group. ** *p* < 0.01 compared with Sham-V group. + *p* < 0.05 compared with SNL-V group.

At baseline, we observed no difference in anxiety-like behavior in the OFT between the different experimental groups. After 4 weeks intervention, (i) relative to the Sham-V rats, the SNL-V rats entered the central area of the open field less frequently, and (ii) relative to the SNL-V rats, the SNL+600GEG rats exhibited a reduction in anxiety-like behaviors, with more frequent of entries into the central area (Figure 2).

### 3.3. GEG Administration Modified Gut Microbiome Composition

**Figure 3:**
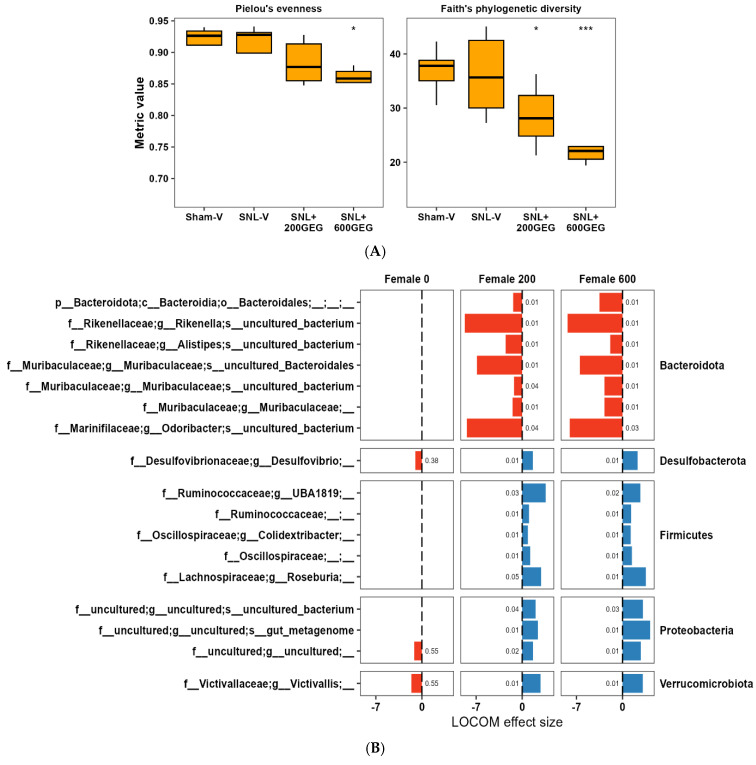
Effects of GEG on gut microbiota. (**A**) Alpha diversity analysis of the microbiome in different groups. Alpha diversity was described using two metrics, one for evenness and one for richness. The Wilcoxon test was used to determine statistical significance. The SNL group was used as a reference group for comparison. * *p* < 0.05, *** *p* < 0.001. (**B**) Compositional analysis of the microbiome ASVs across different groups using LOCOM. Female 0 indicates differences between SNL-V and Sham-V; Female 200 indicates differences between SNL+200GEG and SNL-V; and Female 600 indicates differences between SNL+600GEG and SNL-V. Only ASVs with *p* < 0.05 are reported, and labels indicate FDR-adjusted *p*-values.

We examined GEG administration’s effect on the gut microbiome of rats. Overall, the SNL procedure did not alter alpha diversity of the gut microbiome relative to the sham procedure, but GEG treatment decreased the community alpha diversity, as especially shown by a decrease in species evenness and richness in the SNL+600GEG group (Figure 3A). Figure 3B shows the results of LOCOM. The SNL procedure showed a limited effect on the microbiome composition compared with the sham procedure. The ASVs of Victivallis, Desulfibrio, and an uncultured Proteobacteria were decreased in the SNL group vs. the sham group (*p* < 0.05, but *P*_FDR_ > 0.05) (Figure 3B).

We further examined how the gut microbiome composition reacted to GEG using the same LOCOM approach. Compared with the SNL-V group, both the SNL+200GEG group and SNL+600GEG had similar patterns of gut microbiome composition. We observed a dose effect in several ASVs. The three ASVs decreasing in the SNL animals without GEG administration were Victivallis, Desulfibrio, and an uncultured Proteobacteria ASV, which increased in the GEG-administered groups (*p* < 0.05, but *P*_FDR_ < 0.05). Moreover, several ASVs belonging to *Bacteroidota* decreased with GEG treatments, such as *Rikenella*, *Odoribacter*, and *Muribaculaceae*. In contrast, several ASVs belonging to Firmicutes increased with GEG supplementation, such as *Roseburia* and *UBA1819*.

### 3.4. GEG Administration Modified Fecal Metabolites

**Figure 4:**
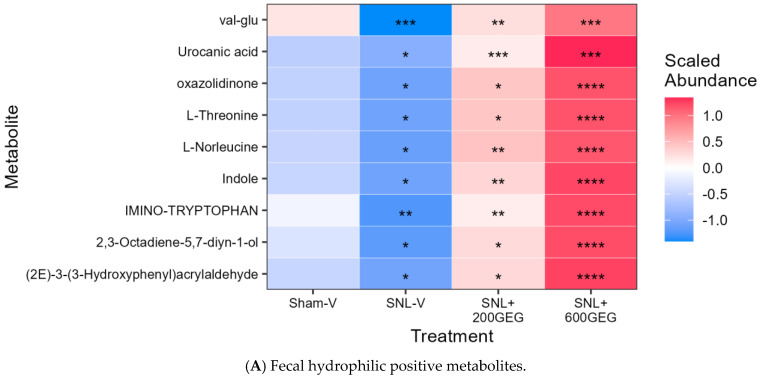

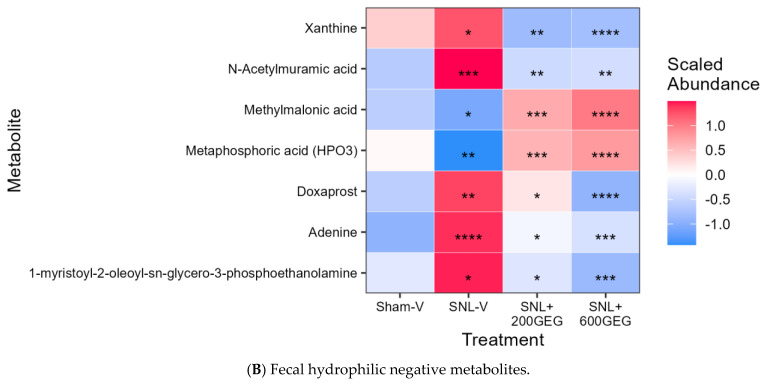
Effects of GEG on fecal hydrophilic positive metabolites (**A**) and negative metabolites (**B**). Data were analyzed by ANOVA on each dataset, followed by the Benjamini–Hochberg procedure for selected compounds with *p* ≤ 0.001 for pairwise comparisons. * *p* < 0.05, ** *p* < 0.01, *** *p* < 0.001, and **** *p* < 0.0001, comparing abundancy of fecal metabolites between groups. Abbreviation: val-glu: valyl-glutamate.

We identified-gut microbiome-derived metabolites that were altered by SNL treatment and reversed by GEG administration in both hydrophilic positive (Figure 4A) and negative metabolites (Figure 4B). In terms of hydrophilic positive metabolites, relative to the SNL-V rats, both SNL+200GEG and SNL+600GEG rats exhibited higher levels of nine metabolites in a dose-dependent manner, namely valyl-glutamate (val-glu), urocanic acid, oxazolidinone, L-threonine, L-norleucine, indole, imino-tryptophan, 2,3-octadiene-5,7-diyn-1-ol, and (2E)-3-(3-Hydroxyphenyl)acrylaldehyde (Figure 4A). With regard to hydrophilic negative metabolites, relative to the SNL-V rats, both GEG-administered rats had higher levels of two fecal metabolites (methylmalonic acid and metaphosphoric acid) and lower levels of five fecal metabolites (xanthine, N-acetylmuramic acid, doxaprost, adenine, and 1-myristoyl-2-oleoyl-sn-glycero-3-phosphoethanolamine).

### 3.5. GEG Administration Modulated the Expression of Neuropathology Genes

**Figure 5:**
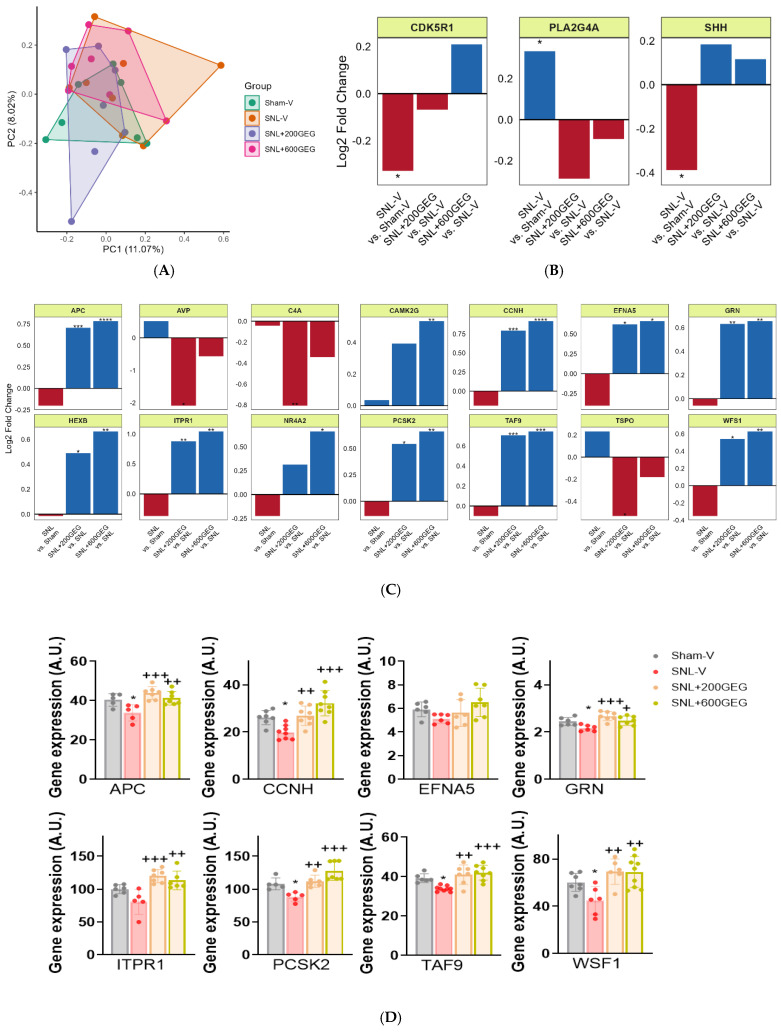
NanoString neuropathology pathway panel analysis of amygdala tissue. (**A**) Principal component analysis of the gene expression profiles of neuropathology-related genes. (**B**) Differential expression analysis between SNL-V and Sham-V. (**C**) Differential expression analysis between SNL+200GEG and SNL+600GEG and SNL-V. * *p* < 0.05; ** *p* < 0.01, *** *p* < 0.001, **** *p* < 0.0001, *t*-test. (**D**) Gene expression levels were assessed by qRT-PCR. * *p* < 0.05 compared with Sham-V group. + *p* < 0.05, ++ *p* < 0.01, and +++ *p* < 0.001 compared with SNL-V group, one-way ANOVA with Bonferroni multiple comparisons tests. Abbreviations: APC: adenomatosis polyposis coli, AVP: arginine vasopressin, C4A: complement C4A, CAMK2G: calcium/calmodulin dependent protein kinase II gamma, CCNH: cyclin H, CDK5R1: cyclin dependent kinase 5 regulatory subunit 1, EPFN5: ephrin A5, GRN: granulin, HEXB: hexosaminidase subunit beta, ITPR1: inositol 1,4,5-trisphosphate receptor 1, NR4A2: nuclear receptor subfamily 4 group A member 2, PCSK2: proprotein convertase subtilisin/kexin type 2, PLA2G4A: phospholipase A2 group IVA, SHH: Sonic hedgehog signaling molecule, TAF9: TATA-box binding protein associated factor 9, TPSO: translocator protein, WFS1: Wolfram syndrome 1 homolog.

The neuropathology panel included 770 genes, which were screened for gene expression and pathways involved in neuropathology (compartmentalization and structural integrity; neuroplasticity, development, and aging; metabolism; neuroinflammation; and neurotransmission) and their modulation by GEG in amygdala tissue. We focused on the amygdala because of its critical role in NP-associated emotional/affective aspects and NP modulation [13,14,15]. We used NanoString nCounter^®^ Neuropathology Panel to examine potential changes. A principal component analysis (PCA) of normalized gene expression profiles indicated similar profiles across all groups (Figure 5A). The SNL procedure induced minor changes in gene expression compared with the Sham-V group, i.e., the expression of 3 genes (out of 770 genes), namely, cyclin dependent kinase 5 regulatory subunit 1 (CDK5R1), phospholipase A2 group IVA (PLA2G4A), and Sonic hedgehog signaling molecule (SHH), was altered (*p* < 0.05) to a small degree (Figure 5B).

Next, we focused on the changes in gene expression induced by GEG. Among the 770 genes examined, 14 differentially expressed genes were confirmed in the comparisons between SNL+200GEG and SNL-V and between SNL+600GEG and SNL-V (Figure 5C), namely adenomatosis polyposis coli (APC), arginine vasopressin (AVP), complement-activation fragment C4A (C4A), calcium/calmodulin dependent protein kinase II gamma (CAMK2G), cyclin H (CCNH), ephrin A5 (EPFN5), granulin (GRN), hexosaminidase subunit beta (HEXB), inositol 1,4,5-trisphosphate receptor 1 (ITPR1), nuclear receptor subfamily 4 group A member 2 (NR4A2), proprotein convertase subtilisin/kexin type 2 (PCSK2), TATA-box binding protein associated factor 9 (TAF9), translocator protein (TSPO), and Wolfram syndrome 1 homolog (WFS1). The majority of gene expression changes were dose dependent, except for AVP, C4A, and TSPO, which showed a greater reduction with 200 mg/kg GEG than 600 mg/kg GEG but did not reach statistical significance in any case.

Next, we analyzed genes with a common signature between the SNL+200GEG and SNL+600GEG groups and those with a dose-dependent response to different levels of GEG administration. Compared with the SNL-V rats without GEG, both GEG-administered rats had eight upregulated genes with a fold change of >0.5 (i.e., APC, CCNH, EFNA5, GRN, ITPR1, PCSK2, TAF9, and WFS1) in the amygdala (Figure 5C). We validated the above findings, except for EFNA5, using qRT-PCR (Figure 5D). This finding implies GEG’s ability, at least in part, to revert the molecular signature of neuropathology in the amygdala of NP rats.

**Figure 6:**
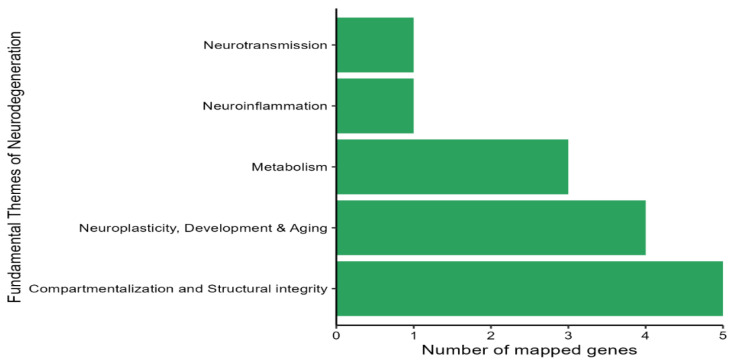
Pathway enrichment analysis using NanoString fundamental themes to map top genes identified as differentially regulated by GEG treatment.

Finally, by mapping the GEG-affected genes to NanoString fundamental themes, five themes were identified: compartmentalization and structural integrity; neuroplasticity, development, and aging; metabolism; neuroinflammation; and neurotransmission (Figure 6).

## 4. Discussion

Our study analyzed GEG’s effects on NP symptoms and the gut (microbiome composition and its metabolites) and brain (neuropathology gene signature in the amygdala) of female rats. The key findings are as follows. Only the high dose of GEG mitigated both NP-associated mechanohypersensitivity and anxiety-affective-like behavior, which agreed with our published works in male rats with SNL [4,17] and high-fat diet plus streptozotocin [5] models of NP. This is the first study to show how GEG administration mitigates NP-induced behavioral changes, modifies gut microbiome composition and metabolites, and reverses the neuropathology gene signature in the amygdala of female NP rats in a GEG dose-dependent pattern. Our findings provide further evidence that GEG could improve NP behaviors via the microbiota–gut–brain axis [17].

In this study, we observed that GEG administration increased the relative abundance of *Firmicutes* and *Proteobacteria* and decreased the relative abundance of *Bacteroidetes* in female NP animals, which matched previous studies in humans showing that ginger decreased the relative abundance of *Bacteroides* in patients with colon cancer compared to those in placebo controls [29,30]. The beneficial effects of ginger on the gut microbiota in female NP rats were also reported in healthy adults [31].

Gut microbiome compositions have been linked to the pathogenesis of pain and anxiety. In this study, GEG administration increased the relative abundance of *Desulfovibrio* and *Ruminococcaceae_UBA1819* in feces of female rats with NP. In a male mouse model of chronic inflammatory pain with anxiety, an increase in *Desulfovibrio* in anxiety-resilient mice suggests that *Desulfovibrio* has beneficial effects on pain resilience and pain-related anxiety-like behaviors [32]. In a chronic constrictive injury-induced NP model in male animals, a decreased abundance of *Ruminococcaceae* was reported in the feces [33]. Similar decreased *Ruminococcus* abundance was also found in patients with spinal cord injury [34]. In the present study, GEG administration mitigated hypersensitivity and anxiety-like behaviors and modified the gut microbiome’s composition (*Desulfovibrio* and *Ruminococcaceae_UBA1819*) in female NP rats, suggesting GEG mitigates NP-associated behaviors, in part, through the modulation of gut microbiota.

Ginger supplementation was associated with a greater abundance of the genera *Ruminococcaceae incertae sedis* and the family *Defluviitaleaceae*, which are known to produce short chain fatty acids (SCFAs), such as acetate and butyrate, in young adults [30]. This aligns with current findings and other studies stating that GEG significantly increases the abundance of SCFAs-producing taxa, e.g., *Ruminococcaceae* [30,35], *Colidextribacter* [5,36,37], *Oscillospiraceae* [38], and *Roseburia* [39,40], in female feces because of its anti-inflammatory properties. *Colidextribacter* has anti-inflammatory effects on the human intestine [37,41]. An imbalance in the gut microbiome, including reduced levels of *Oscillospiraceae*, might play an important role in the progression of inflammatory bowel disease [42] and rheumatoid arthritis [43]. *Roseburia* is a genus of bacteria in the *Lachnospiraceae* family that can help control gut inflammation. *Roseburia intestinalis* produces butyrate, which has been linked to the prevention of inflammation in the intestines and maintenance of energy balance, and it also regulates immune cells and cytokine release [39,44]. A decreased relative abundance of the *Lachnospiraceae* family genus *Roseburia* was reported in chronic pain patients compared with healthy controls [45]. The present study, along with other studies, showed that GEG was associated with decreased abundance of “potential pro-inflammatory” taxa in female rats with NP, such as *Rikenella* [17,46], *Alistipes* [47,48], and *Muribaculaceae* [17,49], offering further support for GEG’s anti-inflammatory effects in mitigating NP symptoms [50]. We noted that GEG’s effect may differ based on sex. In our previous study with NP male rats, administering GEG decreased the relative abundance of *Rikenella*, *Muribaculaceae*, *Clostridia UCG-014*, *Mucispirillum schaedleri*, *RF39*, *Acetatifactor*, and *Clostridia UCG-009*, while it increased the relative abundance of *Flavonifactor*, *Hungatella*, *Anaerofustis stercorihominis*, and *Clostridium innocuum* group [17].

Our observations in this study of GEG’s anti-inflammatory effects on fecal metabolites (e.g., urocanic acid (UCA), oxazolidinone, 3-hydroxyphenyl-acryladehyde, and metaphosphoric acid) in NP females are supported by published studies [51,52]. UCA derivatives had anti-inflammatory capacities in inflammatory bowel disease both ex vivo and in a preclinical mouse model [51]. Treating inflamed colonic tissue with UCA derivatives resulted in reduced proinflammatory IL-6 and IL-8 production and increased anti-inflammatory IL-10 production. UCA derivates decreased the area of inflammation and the number of infiltration neutrophils in mice with colitis. Oxazolidinone is an organic compound with a five-membered heterocyclic ring containing both nitrogen and oxygen; some important variants are used as antibiotics [53]. Based on in vitro and in vivo studies, synthetic oxazolidinone (Linezolid) suppresses infection-induced proinflammatory cytokines [54]. These structural characteristics give 3-hydroxyphenyl-acryladehyde its anti-inflammatory and antioxidant properties [52]. Metaphosphoric acid also has antioxidant traits and has been commonly used to stabilize vitamin C to keep it from oxidizing [55]. The increase in the above metabolites with GEG administration in a female NP model supports our hypothesis that GEG has beneficial behavioral effects in part through the alteration of anti-inflammatory and antioxidant gut metabolites.

Intriguingly, this is the first study to report GEG’s antinociceptive effects in a female NP model through the modulation of gut-microbiome-derived metabolites, as evidenced in increased levels of L-threonine [56,57], indole [58,59], and imino-tryptophan [60,61] using male animals. L-threonine is involved in intestinal health and function and helps maintain dynamic intestinal homeostasis through beneficial effects on the immune system, barrier, gut microorganisms, and intestinal morphology [62,63]. L-threonine (an amino acid) mitigates NP progression by (i) blocking nerve-injury-induced hippocampal TNFα overproduction in animals with chemotherapy-induced NP [56], (ii) enhancing glycinergic postsynaptic suppression of the motor reflex arc in the spinal cord of patients with multiple sclerosis [64], and (iii) changing amino acid metabolism in the brain of rats with diabetic neuropathic pain to decrease analgesic neurotransmitters [57]. Indole-containing compounds have the potential to mitigate pain behaviors in NP mouse models via their anti-inflammatory, antioxidant and antinociceptive properties [58,59]. The anti-NP effects of L-threonine, indole, and imino-tryptophan may be due to improving gastrointestinal integrity and favoring gut microbiome composition. Indole-3-lactic acid, a key molecule produced by *Lactobacillus*, protects against intestinal inflammation and corrects gut dysbiosis [33]. Indoles also contribute to maintaining the intestinal barrier via anti-inflammatory activities, mainly through activating aryl hydrocarbon receptors (AhR) receptors to improve intestinal health in human gastrointestinal disorders [65]. Endogenous tryptophan metabolites, such as imino-tryptophan (a metabolite of the tryptophan–kynurenine pathway), play an important role in gut immune homeostasis in both a mouse colitis model and patients with colorectal cancer [66,67]. An alteration of the microbiota-derived tryptophan metabolism (i.e., a decreased indole/tryptophan ratio) is associated with disrupted intestinal barrier function in the progression of colorectal cancer [67]. Our study highlights potential mechanisms through which microbiome–host interactions, mediated by the previously mentioned microbiota-derived metabolites, may help alleviate NP progression via GEG.

This study is also the first to show that GEG resulted in lower levels of fecal xanthine and N-acetylmuramic acid, which are associated with inflammation and oxidative stress, extending the means by which GEG can counteract NP pain in female rats. Xanthine oxidase is an enzyme that can contribute to NP by promoting inflammation and oxidative stress and macrophage activation [68]. Administration of a xanthine oxidase inhibitor suppressed oxidative stress-induced damage and proinflammatory macrophage activation in male mice with diabetic neuropathy. A positive correlation was found between fecal N-acetylmuramic acid levels and the progression of intervertebral disk degeneration-induced lower back pain in male rabbits [69]. These findings suggest that males and females share certain common characteristics (i.e., anti-inflammation and anti-oxidative-stress) in fecal metabolites associated with NP across the species.

We noted that GEG administration increased the levels of the nonphysiological amino acid L-norleucine in feces. L-norleucine and its derivative 6-diazo-5-oxo-L-norleucine have been studied for their potential pain-relieving effects, particularly in the context of cancer and inflammation, because of their ability to suppress glutamine-mediated inflammation and pain pathways [70,71]. In addition, we observed that the levels of 1-myristoyl-2-oleoyl-sn-glycero-3-phosphoethanolamine in feces were lower in GEG-treated rats. A type of phospholipid, 1-myristoyl-2-oleoyl-sn-glycero-3-phosphoethanolamine can act as a signaling molecule and has been shown to increase intracellular calcium in neurons and induce the release of inflammatory cytokine mediators in immune cells, indirectly increasing pain perception [72]. A reduction in 1-myristoyl-2-oleoyl-sn-glycero-3-phosphoethanolamine levels in the feces of GEG-treated rats could contribute to GEG’s antinociceptive role in NP development.

We do not currently possess thorough knowledge regarding neuroplastic changes in the brain due to chronic pain and the mechanisms behind it, with females in particular being understudied. NP-related neuroplasticity in the amygdala is time- and cell-type-specific [73]. Here, we analyzed neuropathology markers, including genes linked to neuroplasticity, in the amygdala and their modulation by GEG in female rats with NP. In the present study, we identified three genes that were differentially expressed in SNL compared with sham controls (decreased CDK5R1, increased PLA2G4A, and decreased SHH in SNL-L group). PLA2G4A upregulation was consistent with previous work showing elevated PLA2G4A expression in an animal model of spinal cord injury [74,75]. PLA12G4A can release arachidonic acid and other bioactive lipids to promote central sensitization, glial activation, and neuronal excitability in the nervous system [74,75]. Inhibition of spinal secretory PLA12G4A mitigates hypersensitivity and spinal neuronal hyperexcitability by modifying spinal glutamatergic signaling [76]. Studies have shown that SHH signaling plays a critical role after peripheral nerve injury in promoting axonal survival, Schwann cell proliferation, and angiogenesis at the injury site, thereby facilitating nerve healing and functional recovery [77,78]. In this study, GEG showed a slight tendency to downregulate PLA12G4A gene expression in the amygdala of NP rats.

In this study, we observed upregulation of the neuroinflammatory marker genes AVP, C4A, and TSPO in the amygdala of SNL-V rats. AVP upregulates blood–brain barrier permeability, stimulates inflammatory response [79], and induces immunostimulation [80] during brain neuroinflammation in the autoimmune encephalomyelitis (EAE) animal model. We previously reported that AVP in the amygdala contributed to emotional responses and anxiety-affective-like behaviors in male rats in acute pain [81]. C4A activates neutrophiles that increase inflammation and free radicals [82,83], and increased expression of C4A was found in the subventricular zone in a mouse model with schizophrenia-associated phenotypes, contributing to enhanced microglial synaptic engulfment, reduced cortical synaptic density, and behavioral deficits [83]. TSPO is considered as a marker of activated microglia [84]. In this study, GEG administration resulted in the downregulation of C4A and TSPO genes in the amygdala, whereas CCNH gene expression levels were upregulated. CCNH, a protein-coding gene, has been implicated in the resolution of inflammation by promoting the removal of dominant inflammatory cells (i.e., neutrophils) [85]. Thus, the changes in the expression of these genes (C4A, TSPO, and CCNH) might be involved in the inhibitory behavioral effects of GEG in NP.

In this study, GEG administration upregulated the expression of four transcripts (CAMK2G, HEXB, GRN, and NR4A2). Our study presents novel findings that GEG administration significantly increases transcripts of secreted molecules associated with neuroplasticity, development, and aging (i.e., APC, EFNA5, ITPR1, TAF9) as well as compartmentalization and structural integrity (i.e., APC, EFNA5, ITPR1, PCSK2, WFS1), which might suggest GEG’s neuroprotective role in NP. CAMK2G is expressed in most neuronal cells and has a central part in synaptic plasticity of the brain during neurodevelopment [86]. HEXB is specifically expressed in microglia and gives rise to beta-hexosaminidase B, an enzyme that is critical to neuronal development in the spinal cord and brain [87,88]. The GRN gene encodes the protein progranulin, which plays a key role in the function and development of neurons and microglia in the brain. Elevating and/or restoring progranulin levels is an promising therapeutic approach for neurodegenerative diseases [89]. NR4A2 gene is a transcription factor that plays a key role in the development, regulation, and maintenance of dopaminergic neurons in the brain. NP4A2 may have neuroprotective and anti-inflammatory capacities to slow down age-associated memory decline in mice [90]. Increased compartmentalization and structural integrity provide a basis for biochemical reaction and signaling mechanisms [91] and allow neurons to interact functionally and integrate sensory input via gap junctions [92]. For instance, APC is an essential regulator in maintaining the polarity of the radial glial scaffold and is critical for the formation of the cerebral cortex in mammals during brain development [93]. EFNA5 has shown to increase the motility of cortical neurons, which depends on the activity of Src-family kinases in neuron development [94]. The ITPR1 gene is important for regulating cellular calcium distribution in the brain, particularly in the cerebellum, during the development and function of brain tissues [95]. TAF9 is also expressed in neurons and is part of a cluster of genes associated with synaptic function in the brain [96]. Within the regulated secretory pathway of neuroendocrine cells, PCSK2 is a prohormone activator and a neuropeptide precursor [97]. Therefore, increased transcription of these above genes due to GEG administration suggests GEG’s potential beneficial effects in NP through neuroprotection.

We noted that GEG administration increased gene expression associated with pain neurobiology (WFS1, PCSK2, and TAF9) in SNL rats. The role of WFS1 is to maintain endoplasmic reticulum homeostasis and calcium homeostasis within cells [98]. The WFS gene is a critical regulator of neuronal function and excitability, which is important for pain processing [99,100,101]. WFS1 dysfunction triggers endoplasmic reticulum stress, leading to the release of inflammatory molecules and other factors that can exacerbate pain [102]. The PCSK2 gene plays an important role in pain neurobiology by cleaving neuropeptide precursors into their active forms and potentially increasing opioid receptor levels, which are important for pain modulation [103]. TAF9 is associated with peripheral and central sensitization, key aspects of NP [56]. While direct evidence linking high TAF9B expression to pain is limited, TAF9′s role in neuronal development and gene regulation suggests a potential connection. TAF9′s function is like that of a transcriptional regulator in the RNA polymerase II complex to transcribe genes into RNA, suggesting TAF9 could influence the expression of genes involved in neuronal activity, inflammatory responses, and possibly pain transmission [104]. Understanding the role of TAF9 in neuronal development and pain signaling could lead to new therapeutic strategies for pain management, including for NP [96].

The limitations of this study include the following: (i) because of the small amount of amygdala tissue, we selected only six samples per group for neuropathology gene profiling, and (ii) while the RNeasy Mini Kit is a good starting point for RNA extraction, it is recommended to check RIN scores to ensure that RNA samples are of high quality for RNA sequencing.

## 5. Conclusions

Oral administration of GEG to NP female rats (i) decreased pain and pain-associated anxiety-like behavioral responses, (ii) reverted NP-related changes in gut microbiome composition (increased abundance of phyla *Bacteroidota*, *Firmicutes*, and *Verrucomicrobiota*, (iii) modified microbiome-derived metabolites (increased levels of val-glu, urocanic acid, oxazolidinone, L-threonine, L-norleucine, indole, imino-tryptophan, 2,3-octadiene-5,7-diyn-1-ol, (2E)-3-(3-hydroxyphenyl) acrylaldehyde, methylmalonic acid, and metaphosphoric acid, as well as decreased levels of xanthine, N-acetylmuramic acid, doxaprost, adenine, and 1-myristoyl-2-oleoyl-sn-glycero-3-phosphoethanolamine), and (iv) reversed neuropathological signature genes (upregulated APC, CCNH, EFNA5, GRN, HEXB, ITPR1, PCSK2, TAF9, and WFS1, as well as downregulating AVP, C4A, and TSPO) in the amygdala. A response to limited GEG dosage in the gut microbiome effect was observed, while GEG’s effects on fecal metabolites were dose dependent.

## Figures and Tables

**Table 1:** List of primers.

Gene	Forward	Reverse
APC	5′-AGC AAG TTG AGG CCC TGA AGA-3′	5′-ATA CTT CCC TGC AGC TGC TTA-3′
CCNH	5′-AGC CAC CCA GAT CTG AAG AAG TT-3′	5′-GTC ATC GTC CGT CCA CTC CT-3′
EFNA5	5′-GGA CCG CTG AAG TTC TCG GA-3′	5′-CGA AAA CAC GAT CAC GAA CAC CT-3′
GRN	5′-CTG GAG CTG ACC GCC AGA TG-3′	5′-GTC AAG GCA GCA GGC AAC AG-3′
ITPR1	5′-TTG GAA AAT GCC GAG CTG CC-3′	5′-GGG GTG GAC TTG GTT CAA GC-3′
PCSK2	5′-ACG TTC AGC AAC GGG AGG AA-3′	5′-AAG CCA ATG CAA ACA CGC CA-3′
TAF9	5′-TTC CGA CA TCC TGC TCA CCG-3′	5′-CATBCTG TGC TC TTT CGG CA-3′
WFS1	TGC TGG AGC GTC TAG TGA GC-3′	5′-CTT CTG GCG TAG TGG CAG GT-3′
β-actin	5′-ACA ACC TTC TTG CAG CTC CTC C-3′	5′-TGA CCC ATA CCC ACC ATC ACA-3′

Abbreviations: APC: adenomatosis polyposis coli, CCNH: cyclin H, EFNA5: ephrin A5, GRN: granulin, ITPR1: inositol 1,4,5-trisphosphate receptor 1, PCSK2: proprotein convertase subtilisin/kexin type 2, TAF9: TATA-box binding protein associated factor 9, and WFS1: Wolfram syndrome 1 homolog.

## Data Availability

The raw sequencing data (BioProject access number PRJNA935472) of 16s RNA sequencing (gut microbiota) was deposited in the National Center for Biotechnology Information BioProject database. The original contributions presented in this study are included in the article. Further inquiries can be directed to the corresponding authors.
